# The ripple effect, silence and powerlessness: hidden barriers to discussing suicide in Australian Aboriginal communities

**DOI:** 10.1186/s40359-022-00724-9

**Published:** 2022-02-07

**Authors:** Todd R. Heard, Katherine McGill, Jaelea Skehan, Bronwyn Rose

**Affiliations:** 1 Department of Health, Hunter New England Local Health District, 72 Watt Street, Newcastle, NSW 2320 Australia; 2 School of Medicine and Public Health, University of Newcastle, Newcastle, NSW Australia; 3 Faculty of Business, Justice, and Behavioural Sciences, Charles Sturt University, Newcastle, NSW Australia

**Keywords:** Aboriginal, Suicide, Attitudes, Barriers to discussing suicide, Qualitative, Community consultation, Prevention, Culturally appropriate mental health care, Bereaved by suicide

## Abstract

**Background:**

Suicide is one of the leading causes of death for Aboriginal Australians. There is an urgent need to actively engage with Aboriginal communities to better understand these issues and to develop solutions together to prevent deaths by suicide in Aboriginal communities.

**Methods:**

Utilising a qualitative, thematic, cross-sectional design, we conducted focus groups in three communities in the Hunter New England area in New South Wales (Australia) to explore the perceptions and views of Aboriginal participants in relation to discussing suicide.

**Results:**

The key themes found to influence discussions about suicide in Aboriginal communities included the sense that suicide is a whole of community issue, the ripple effect of suicide deaths, silence about suicide and the impact of this silence, and being powerless to act. Participants described a reluctance to have discussions about suicide; feeling they had limited skills and confidence to have these sorts of discussions; and multiple and interrelated barriers to discussing suicide, including shame, fear and negative experiences of mental health care. Participants also described how their experiences maintained these barriers and prevented Aboriginal Australians from seeking help in suicidal crises.

**Conclusion:**

Future initiatives should address the interrelated barriers by supporting Aboriginal people to build skills and confidence in discussing suicide and mental health and by improving access to, and the experience of, mental health care and psychosocial and community-based supports for Aboriginal Australians. We suggest trying to address any one of these factors in isolation may increase rather than decrease suicide risk in Aboriginal communities.

## Background

In Australia, suicide is one of the leading causes of preventable death for Aboriginal people [1, 2]. Aboriginal Australians are more likely to die by suicide than non-Aboriginal Australians, with rates for Aboriginal men increasing at a rate greater than that for Aboriginal women [3]. Age-specific rates of suicide are highest among Aboriginal people aged 15–34 years, with deaths by suicide being the leading cause of death for this age group [3]. There is also growing policy commitments and attention to Aboriginal suicide prevention within Australia, including the development of a renewed National Aboriginal and Torres Strait Islander Suicide Prevention Strategy [2] and the establishment of a Centre of Best Practice in Aboriginal and Torres Strait Islander Suicide Prevention. These initiatives prioritise the importance of identifying interventions and programs that work for Aboriginal people and the need to grow the evidence base. 

This is because there is relatively limited published work that explores the factors that may serve to increase or decrease the risk of suicide in Aboriginal communities specifically. A number of demographic, health and social factors, including experiences of racism, trauma, grief and loss, highly interrelated and connected relationships and lack of autonomy, are collectively thought to contribute to increased risk of suicide for Aboriginal people [4–8]. Whilst it is not known how these risk factors specifically translate into more deaths by suicide, their impact is thought to be cumulative in nature, with individuals and communities who experience more than one risk factor being at greater risk of suicide [5].

There is also limited published research exploring the effectiveness of approaches to reduce the risk of suicide in Aboriginal communities. Using a systematic review methodology, Ridani et al. [9] concluded that research in this area was limited and often lacked sufficient evidence to demonstrate an intervention’s effectiveness in reducing the risk of suicide at an individual or at a community level. Ridani et al. [9] and Nasir et al. [10] both describe a tendency for existing non-Aboriginal suicide prevention programs to be modified for use in Aboriginal communities, often without considering the appropriateness of the program for Aboriginal people. They similarly highlight that effective approaches to suicide prevention in Aboriginal communities are more likely to focus on building community-level protective factors, such as community connectedness, belongingness and cultural pride, rather than focusing on an individualistic approach, which is more common in non-Aboriginal programs [9, 10]. In recognition of the importance that culture plays in suicide prevention, it is now not considered sufficient to simply modify non-Aboriginal approaches. Rather, community consultations to hear the views of grassroots people and people with a lived experience of suicide is considered a necessary part of the development process for suicide prevention interventions [11] and it is recommended that suicide prevention programs should specifically seek to complement Aboriginal cultural beliefs and practices [10–12].

To our knowledge, only four qualitative studies [7, 10, 13, 14] have been conducted and published utilising a community consultation methodology to explore the views of Aboriginal Australians about what impacts discussions about mental health and suicide. The findings of these studies highlighted that attitudes towards discussing mental health and suicide vary, with some participants being strongly in favour of discussing suicide and others having no intention to discuss suicide because they believe it is an individual issue and not the business of others [7, 10, 13, 14]. All studies identified that fear, shame, mistrust and poor experiences of mental health services negatively impacted community attitudes towards discussing mental health and suicide and resulted in ambivalence about encouraging access to mental health services for Aboriginal people in a suicide crisis [7, 10, 13, 14].

Given the growing rate of Aboriginal suicide in Australia [3], there is an urgent need to continue to build on this work, particularly with regard to developing a better understanding of the factors that drive and maintain barriers to discussing mental health and suicide and those that affect the help-seeking behaviour of Aboriginal people when experiencing mental health difficulties or in a suicide crisis. Furthermore, while the existing studies were conducted in three different geographical locations, Aboriginal communities are diverse, and it is important to understand the views of a broad cross-section of Aboriginal communities to allow for a more detailed and comprehensive examination of driving factors, particularly to inform the development of culturally specific Aboriginal suicide prevention initiatives [13].

Consistent with this need, the aim of this study was to build on previous published work [7, 10, 13, 14] by conducting a qualitative community consultation to explore the perceptions and views of a broad cross-section of Aboriginal communities in NSW in relation to discussing suicide.

## Method

This study used a qualitative, thematic, cross-sectional design with a focus group methodology. A project reference group was established to oversee and support the cultural appropriateness of the study and included key stakeholders working in the area of Aboriginal health and Aboriginal social and emotional wellbeing (SEWB) services in the local health district area and Aboriginal people from key representative bodies for Aboriginal people in the study area.

### Participants

Three Aboriginal communities in NSW covering diverse geographical regional areas, including a major city, inner and outer regional and remote areas [15] and including the Aboriginal lands of the Awabakal, Kamilaroi and Biripi people, were considered eligible to participate in the study. Eligible communities were located in the Hunter New England area, had access to a shared publicly funded adult mental health service, an active Aboriginal Community Controlled Health Organisation (ACCHO) with an Aboriginal SEWB team and had existing cultural, kinship and community links between and within communities. Aboriginal people aged 18 years and over were eligible to participate in the study. All eligible participants experiencing acute symptoms of mental illness at the time of the study and those who had been bereaved by suicide in the past 12 months were not eligible to participate in the study.

### Recruitment

The chief executive officers of the ACCHO in each eligible community were individually approached by a member of the project reference group to participate in the study. All approached communities agreed to participate in the study. Eligible participants were recruited via a convenience advertising (posters located at participating services) and snowballing methodology. The study partnered with the ACCHO and Aboriginal SEWB workers employed by publicly funded local health district Aboriginal SEWB services to assist with the recruitment and screening of potential participants.

### Procedure

Prior to participating in the study, potential participants were screened for eligibility. Any community member deemed ineligible to participate was provided with contact information for Aboriginal SEWB services for ongoing support. If eligible to participate in the study, participants were invited to attend a focus group in their local community.

Three focus groups were conducted in confidential spaces suggested by the participating Aboriginal community. Focus groups were led by two facilitators who had a strong working knowledge of the risks and barriers associated with discussing suicide in Aboriginal communities and always included one Aboriginal psychologist. In addition, each focus group was supported by an independent Aboriginal mental health professional who provided support to anyone who became distressed.

A total of 23 participants participated in three focus groups conducted at three locations in the Hunter New England Area of NSW. Participant numbers were comparable across the groups (Awabakal *n* = 8, Biripi *n* = 7, Kamilaroi *n* = 8).

The focus groups followed a semi-structured format with the same questions being asked regardless of location. The focus group questions included:

1. How important is it to talk about suicide in the community?
2. What conversations about suicide are already occurring in the community?
3. What things do you think make it hard for people to talk about suicide?
4. What things do you think would make it easier for you to talk about suicide?

Whilst semi-structured in nature, the focus group methodology supported an inductive exploration of participants’ thoughts, opinions and beliefs about the topic and encouraged discussion, clarification and understanding [16]. The focus groups were audio-recorded for the purpose of transcribing; they lasted for 108, 113 and 140 min and occurred over a period of three consecutive months. Consistent with cultural expectations, the audio-recordings were destroyed following transcription. Participants were informed that they were unable to review the transcripts due to confidentiality, but if possible their personal contributions would be removed upon request.

### Data analysis

Focus group data was stored and analysed using NVivo, a qualitative data analysis computer software program [17]. The analysis was conducted by an Aboriginal community member/researcher (TH) and a non-Aboriginal researcher (KM) with expert knowledge of suicide, mental health and qualitative methodologies. The data was analysed using a thematic approach that aimed to identify themes representing participant views and perceptions across the data set. Thematic analysis was conducted using a hybrid approach whereby: data was initially analysed based on a deductive methodology with a pre-determined coding framework based on the research questions and cited literature; and with an additional inductive methodology to ensure additional salient codes were included in the analysis. The hybrid approach to qualitative methodologies is consistent with Fereday and Muir-Cochrane’s approach to thematic analysis [12]. Further, the current analysis is guided by and extends the findings of previous research and allows for new or emerging themes given the reviewers specific experience in mental health and suicide in Aboriginal communities.

The initial stage of the analysis involved both researchers reviewing the deductive code set and considering emerging themes based on an inductive methodology. Once initial coding was complete, the researchers met to discuss the use and definition of deductive codes and to discuss emerging themes and to reach agreement on their scope and meaning. The remaining transcripts were reviewed using the final combined code set (Appendix); any similarities and differences were discussed until agreement was reached.

## Results

Four broad themes were identified as underpinning the responses to the focus group questions. These included:

1. Suicide is a whole community issue;
2. The ripple effect of suicide;
3. Silence and its impact; and
4. Being powerless to act.

Whilst there were discrete differences in focus group responses across geographical regions, age and gender, these core themes were apparent in every focus group. The key features of each of the themes and their relationships with subthemes are discussed below.

### Suicide is a whole community issue

All participants spoke about how important it was to discuss suicide in Aboriginal communities. Participants stated it was important to ‘get out there and not be ashamed to talk about suicide’ (Kamilaroi, Speaker 21), particularly because of the perceived increase in and personal impact of suicide in Aboriginal communities. Despite identifying it as being important, the overwhelming majority of participants stated discussions about suicide are not ‘really [occurring] in Aboriginal communities’ (Biripi, Speaker14). In the small number of cases where such discussions were occurring, they were described as being led by community members with experience in Aboriginal SEWB -type positions.

‘I mean I talk about suicide at work, that’s the only time.’ (Kamilaroi, Speaker 55).

‘It’s not about what’s hard, it’s not discussed in our communities.’ (Biripi, Speaker 20).

**‘**I think it is the elephant in the room, no one wants to talk, acknowledge the fact that suicide exists, but it’s such a horrible thing to think about, that you’d rather not think about it.’ (Kamilaroi, Speaker 10).

A small number of participants reported that discussing suicide was not always helpful. Some viewed suicide as an individual decision, believing that ‘no matter what you say or do it’s not going to prevent them from committing suicide’ (Kamilaroi, Speaker 47). One respondent who shared their lived experience of a suicide attempt spoke about their anger towards the person who got them to the hospital thinking ‘how dare you think you can tell me what I can do with my life’ (Kamilaroi, Speaker 15). These comments highlighted the sensitivity needed to navigate discussions about and responses to suicide crises.

### Ripple effect

In each focus group, participants shared personal stories of the perceived community consequences of not discussing suicide, particularly the potential of amplifying the ‘ripple effect’ of trauma experienced in Aboriginal communities following a death by suicide. Peoples’ own lived experiences appeared to motivate many participants to advocate for why communities needed to discuss suicide, as it was seen as being a way to prevent further deaths (trauma) and to ensure that other families and communities do not have to experience the ‘long-lasting’ (Kamilaroi, Speaker 55), cross-generational effects of losing a loved one to suicide.

‘Suicide has a ripple effect. There needs to be discussions and education about how long the ripple effect continues for.’ (Kamilaroi, Speaker 55).

‘People are left behind. They’re also saying “I should have gone there that day. I should have done, why didn’t I do?” I think that is the hardest thing the community left behind are left to clean that and the guilt is huge, [a] mammoth amount of guilt.’ (Kamilaroi, 27:20).

‘I've been aware of instances where siblings have [ended their life by suicide] 12 months later because it solved their sibling’s problem - so that’s what I will do too. So then the family loses two.’ (Kamilaroi, Speaker 20)

‘The grieving process actually still continues and then they still self-medicate and then other problems arise and then that family member ends up in Mental Health, so it's just an ongoing process where they're just following each other.’(Biripi, 44:43).

The ripple effect of trauma following a suicide death was described by participants as including initial grief at the time of the loved one’s death that was felt by the whole community; a sense of strong support and connection at the funeral and for a period following; a sense of isolation and ‘silence’ following the funeral when people returned to their own lives; and in some cases an experience of a cascade of negative events in the family that were managed in ‘silence’. One participant stated that:

“Families are forgotten after a month, and in honest truth, it’s just like a burial. Someone dies in the community, everybody comes to your home for a whole month and all of a sudden they go away and they think and they say. It’s part of life, get on with life” (Biripi, Speaker 66).

The negative events experienced by bereaved families included family units breaking down; disruptions in education, employment and community participation; and in some cases the onset of substance use or mental health issues. The impact of these negative events was also felt by the community at large, with subsequent negative events experienced as additional trauma for the broader family and community.

‘When you lose kids, it’s very, very damaging to your own self. Let alone what happens to the community and how many people come up behind you.’ (Biripi, Speaker 38)

‘Because you want to die and be with them. It doesn’t matter how you die you just want to be with them.’ (Kamilaroi, Speaker 26).

‘All the left-over hurt and grief and misplaced anger, where is it gonna go, and the people who were left behind, everyone is hurting.’ (Kamilaroi, Speaker 55).

**Fig. 1:**
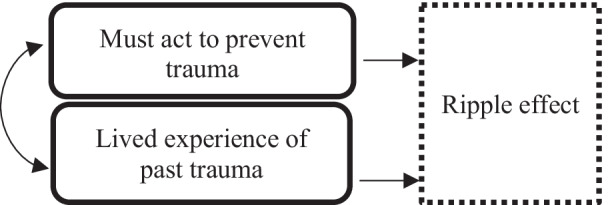
Drivers of the ‘ripple effect’ in Aboriginal communities

In short, the ripple effect captures participants’ experiences of the impact of a suicide as being immediate and long-lasting, the impact as affecting not only those directly or immediately connected to the person but also the broader community, and the sense that the event could initiate a chain of other challenges. The drivers of the ‘ripple effect are summarised in Fig. 1.

### ‘Silence’ and its impact

**Fig. 2:**
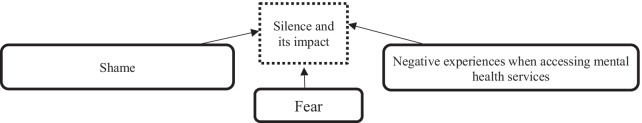
Drivers of ‘silence’ and its impact

The drivers of ‘silence’ and its impact are summarised in Fig. 2. Participants frequently spoke of a ‘silence’ around suicide in their communities, including for community members who had been bereaved by suicide. Participants described how ‘silence’ resulted in family and community members feeling unable to share their experience of losing a loved one to suicide. This resulted in people bereaved feeling isolated from their informal systems of support, feeling stuck and unsupported in their experience of grief and loss and feeling unable to break the ‘silence’ within the family given that in the past it had resulted in family turmoil. One participant reported that their family and community had not talked about a loved one who had died by suicide for ‘16 years, 17 years’ (Kamilaroi, Speaker 55)*.* This silence had caused the participant a great deal of sadness; they felt that the memory of their loved one had been lost and that any attempt to discuss this resulted in a breakdown in family relationships and the onset of or relapse into substance use for some family members. The ‘cost’ of silence was described by many participants. One participant stated:

No one in my family wants to talk about it, that’s the first conversation I think that we have had about my [family member] in 16 years, 17 years, and my father’s response was ‘oh it’s something that you gotta get over’, my mother’s response- wasn’t even a response. Sixteen years and haven’t talked about it, haven’t talked about my cousin, my [family member], we don’t talk about it, we don’t talk about anyone. (Kamilaroi, Speaker 55)

#### Shame

**Fig. 3:**
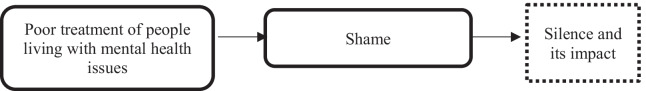
Drivers of shame in terms of discussing suicide in Aboriginal communities

The shame felt by participants and people in the community when discussing suicide and poor mental health was reported by participants as being a factor that prevented discussions about suicide and mental health in their community. The feeling of shame experienced by participants was largely described as being associated with having witnessed the poor treatment of people living with mental health issues in their community. Participants reflected on their own experiences and reported that people living with mental health issues were often isolated in their community; were not provided with the support they needed from community members; were often labelled as ‘womba or whatever you want to call it’ (Biripi, 10:25) (meaning crazy) by other community members; and that the police would often be called to take a person to hospital. One participant acknowledged that ‘there’s no getting away [from the shame], it doesn’t matter how far outside of the country you go, you still have contact with those people, there’s that shame factor’ (Kamilaroi, Speaker 43). Participants felt that if people were to discuss suicide in their community they too would be treated poorly, and this would bring shame to them and their family. ‘Community members that suffer mental health issues, when they go off, people close their doors, they just ring the police’ (Kamilaroi, Speaker 15). The drivers of ‘shame’ in terms of discussing suicide are summarised in Fig. 3.

#### Fear

**Fig. 4:**
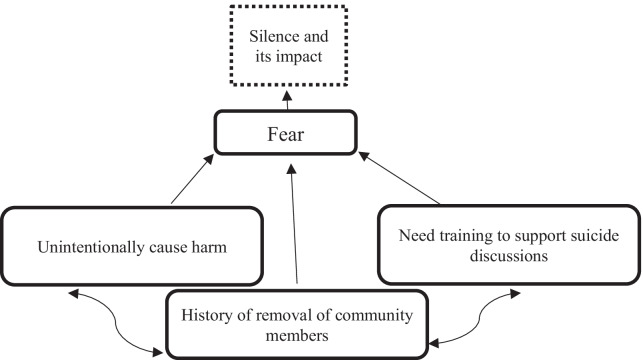
Summary of the drivers of fear in terms of discussing suicide

According to Fig. 4, participants reported being ‘really scared to have [mental health and suicide] discussions with people’ (Awabakal, Speaker23) because they feared the conversation could cause harm to the individual or the community, or that they would not be able to provide appropriate support to a community member if they had the conversation. In their responses, participants described being fearful that the conversation would put ‘the idea in the person’s head’ (Awabakal, Speaker 37), would in some way ‘glorify’ (Awabakal, Speaker 31) or ‘normalise’ (Awabakal, Speaker 31) the idea of suicide, that it would in some way be ‘overstepping the boundaries’ (Awabakal, Speaker 12) of what was right and wrong to say, that it could potentially trigger ‘unresolved grief and loss issues’ (Awabakal, Speaker 34) by bringing up ‘stuff that they are not ready for’ (Awabakal, Speaker 34) that might ‘push them over the edge’(Biripi, Speaker 22). Participants reported that these fears often resulted in them not talking about suicide even when it may have been needed.

Furthermore, many participants described being afraid to discuss suicide given that people who had spoken of having mental health issues or of suicide in the past had been forcibly removed from their community. They described personally witnessing community members ‘that suffered [from a mental health issue] being placed away, out of sight, out of mind’ (Biripi, Speaker 20) in mental health facilities far away from the community and reported being afraid of this occurring to other community members. Participants reported that once placed in these institutions, the community member/s would ‘never come back to the community’ (Biripi, 11:02). The fear of being removed from the community was identified as a potent factor in preventing people from having discussions about suicide in their communities.

Despite being fearful, the participants reported a strong desire to face the fear and to have more open discussions about suicide and mental health in their community. They spoke of wanting access to information and training and resources to build their skills and confidence to safely have these discussions. This included recognising ‘the signs and symptoms of suicide’ (Biripi, 43:39) and ‘general knowledge of mental health’ (Biripi, Speaker 64); support to understand ‘what they can say, because it’s hard to know what to say to somebody’ (Kamilaroi, Speaker 5) if they recognise any signs and symptoms in a community member; and guidance about how to specifically support a person who is thinking of suicide, information about support services and what to say following a death by suicide in the community.

I think sometimes too many people feel that they might be putting the ideas in [someone’s] head by talking about it. ‘I shouldn’t say this because what if I start thinking along that line and that’s what they do’. That’s where the discomfort comes from. (Awabakal, Speaker 37).

‘Some family members won’t even talk to the family and they’re frightened to talk to them because they don’t want to push them over the edge.’ (Biripi, Speaker 22).

#### Negative experiences when accessing mental health services

Negative experiences when accessing mental health services were reported by most participants as another factor that prevented discussions about suicide. Negative experiences were grouped into the following sub-themes: generally feeling not supported by mainstream services; personal experiences of poor follow-up and engagement in support; having experienced no involvement in the mental health treatment and discharge decisions that affected their family and community; having received culturally inappropriate care; and having experienced or witnessed racism when interacting with health services.

**Fig. 5:**
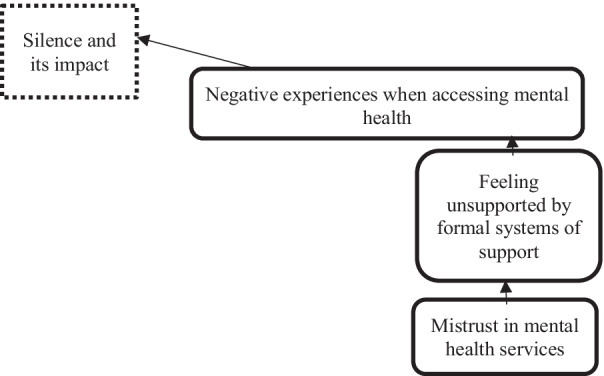
Drivers of negative experiences when accessing mental health services in Aboriginal communities

As summarised in Fig. 5, these factors contributed to the development of ‘mistrust’ between the Aboriginal communities and mental health services and a general reluctance to discuss suicide, given that community members did not feel they could turn to mental health services for reliable or helpful support when a person was suicidal.

‘You’ve got to build up trust up in the hospital.’ (Biripi, 24:03).

‘You’re not educating the family on what they’re medicating with. You’re not educating us. You know, what are the symptoms? What are the signs? You know, and things like that.’ (Biripi, Speaker 49).

‘So a lot of people get ridiculed if you show too much attention on, you know, getting Aboriginal people where they need to be within their health.’ (Biripi, 27:37).

‘It’s that our people for too long have been stigmatised and discriminated against.’ (Biripi, Speaker 62).

Consistent with participants’ experiences of ‘silence’ around discussing suicide in families and communities, they also described experiencing another type of silence when attempting to access mental health services. These services were described as frequently having failed to connect with those bereaved by suicide, those in a suicide crisis or those with mental health issues in a timely way, if at all, with the impact being that community members were turned away by mental health services with no follow-up support. This left already vulnerable Aboriginal community members feeling lonely and not able to reach out for support from either their informal support (family and community) or formal support (mental health services) systems.

‘It could be families out there that aren’t getting support [that’s what we are talking about]. There needs to be some sort of discussion and education around what happens to the family unit when someone does decide to leave and the ripple effect and how long that ripple effect continues on for, just makes the rift wider and wider.’ (Kamilaroi, Speaker 55)

‘But our biggest problem there with mental health is there’s no support up there, there is no support at the hospital.’ (Biripi, Speaker 28).

‘To contact the helpline and then you don’t get help, you go back to ED and you don’t get help, and then you go to mental health and you don’t get help.’ (Biripi, Speaker 55).

‘My baby [family member] died at least 15 years ago, I’m still waiting to get into community health … I mean how long does it take to get me in when someone has died?’ (Biripi, Speaker 26).

### Powerless to act

**Fig. 6:**
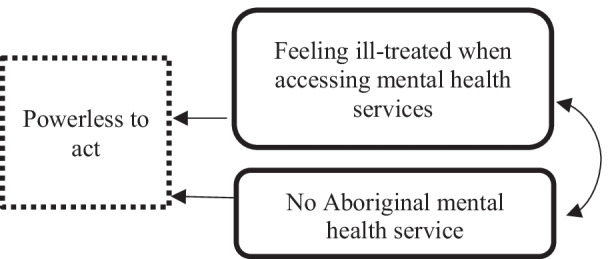
Drivers of being ‘powerless to act’ in terms of suicide in Aboriginal communities

The combined impact of the ripple effect, silence around suicide and people’s poor experiences when accessing mental health services manifested in participants reporting a sense of being ‘powerless to act’ when worried about someone and unable to call on meaningful support from others (in the community or mental health services). Other specific factors reported by community members that contributed to the theme of being ‘powerless to act’ are summarised in Fig. 6.

#### Feeling ill-treated when supporting a community member to access mental health care

In the face of the barriers to accessing mainstream mental health services, participants described how they and others in the community had at times no choice but to engage with a mainstream mental health service when trying to support an Aboriginal community member who was actively suicidal or acutely unwell. When forced to access existing services, participants reported feeling like the system would often treat them like a ‘troublemaker’ (Biripi, Speaker 55) or would at times call the ‘police on them’ (Biripi, Speaker 55*)* when all they were trying to do was support a friend or family member. The persistent negative experiences reported by participants when attempting to access support left some participants feeling powerless and like ‘they didn’t even have the right to good respectful health care’ (Biripi, Speaker 62) and that ‘you get knocked down and you just can’t possibly get back up again’ (Kamilaroi, Speaker 62). The powerlessness described by participants extended to having no confidence that if they chose to make a complaint about their treatment that it would be treated seriously and recognising that if they did make a complaint about the service ‘Where [were they] going to go? There isn’t another service for over one hundred kilometres.’ (Kamilaroi, Speaker 76).

#### No Aboriginal-specific mental health/suicide service

The sense of powerlessness in discussing suicide and being able to support people during a suicide crisis appeared to be amplified by the absence of a ‘trusting’ relationship with mental health services and the absence of Aboriginal-specific mental health services. The majority of participants reported feeling that there was no Aboriginal-specific service or no one in their community that Aboriginal people could sit down and have a ‘talk with to steer them in the right direction’ (Kamilaroi, Speaker 16) or someone that ‘they can identify with and they can feel safe with and they will open up to’ (Kamilaroi, Speaker 16). Participants reported that having access to Aboriginal-specific supports in their community was important because community members ‘especially young ones, they won’t open up to [just] anyone, they won’t [just] go to anyone’ (Kamilaroi, Speaker 26) and the community had felt in the past that they were not understood by counsellors who lacked an ‘understanding [of] where I am coming from, my way of thinking and their training is totally wrong with working with Aboriginal people’ (Biripi, Speaker 22). Participants reported that improving their access to an Aboriginal-specific mental health service and addressing the limited existing capacity of mental health services to provide culturally appropriate care and incorporating more Aboriginal workers or Aboriginal mental health clinicians would be appropriate ways to address the issues.

### Thematic summary

**Fig. 7:**
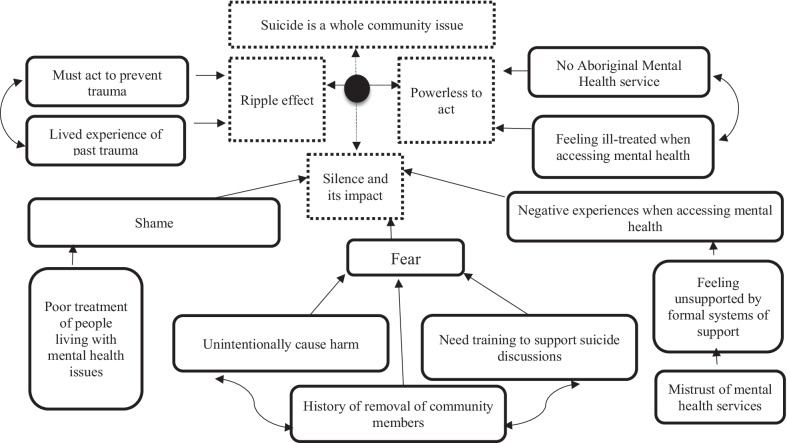
Summary of thematic analysis

As seen in Fig. 7, these themes and subthemes were related to and interacted with each other; with the four main themes (suicide as a whole of community issue, the ripple effect of suicide, silence and its impact, and being powerless to act) specifically tied to one another. It is also important to note that the connection points between these themes and subthemes reflect the ones most frequently identified and with the strongest ties. In short, the themes were tied to each other and experiences/perceptions of one influenced experiences/descriptions in another highlighting the complex interplay of factors impacting suicide discussions within these communities.

## Discussion

The primary purpose of this study was to conduct a qualitative investigation exploring the perceptions and views of Aboriginal people regarding discussing suicide in a broad cross-section of communities in the Hunter New England region of NSW. Four key themes were identified as underpinning responses. Specifically: suicide is a whole community issue, the ripple effect of suicide, silence and its impact and being powerless to act. While the findings are broadly consistent with those of previous studies [5, 7, 10, 13, 14], this study explored in more detail what contributes to these perceptions, highlighting the individual and community factors that drive and maintain known barriers to discussing suicide in Aboriginal communities. These themes and factors are discussed below.

First, the findings highlight that despite participants voicing strong beliefs about the importance of discussing suicide in Aboriginal communities, such discussions were not identified as routinely occurring. This outcome is particularly concerning given the significant prevalence and impact of suicide in Aboriginal communities, and it provokes an urgent call to action for the Government and Aboriginal communities to work together to provide community resources and training (gatekeeper training), as proposed by Nasir et al. [10]. The aim of such training is to build the skills and confidence of Aboriginal communities to have a general understanding of mental health and wellbeing; to recognise the signs and symptoms of suicide and mental illness; to feel able to respond and know what to say when they recognise the symptoms of poor mental health and/or suicide; to support help seeking for community members experiencing a suicide crisis; and to have safe community discussions following a death by suicide. Building these skills is consistent with the call for greater self-determination opportunities and locally developed and led responses as identified as important in the consultation for Aboriginal and Torres Strait Islander Suicide Prevention Evaluation Report [19].

Second, participants described how the barriers of fear, shame and negative experiences when accessing mental health services were responsible for maintaining a ‘silence’ around suicide within their communities. These barriers are consistent with those identified by Vicary and Westerman [7], Deanne et al. [14] and Skehan, Garvey and Scott [20] and highlight a commonality of experiences across different Aboriginal communities. However, we have been able to extend the understanding of how ‘silence’ itself (as a behaviour and a system experience) may perpetuate the cycle beyond what has been described in previous studies. The consistency of findings across studies suggests that future initiatives should specifically seek to address fear and shame as a way of helping Aboriginal people find a voice to discuss mental health and suicide. It also simultaneously highlights the need to prioritise work that addresses the poor experiences with mental health care described by the Aboriginal participants across the studies. In fact, the findings of this study suggest that tackling one without the other (i.e. fear, shame or poor experiences of mental health care) will be ineffective in rendering real change in terms of discussing suicide in Aboriginal communities.

Third, almost all participants reported poor experiences of care when accessing mental health services. They reported having experienced a lack of appropriate support from mental health services when in need and described a perception that mental health services were knowingly or unknowingly using their privilege and power to minimise the needs of Aboriginal people and prevent access to mental health care. These types of experiences are consistent with definitions of systemic (institutionalised) racism, which refers to practices that control or prevent access to a community resource (such as mental health care) resulting in unequal access to a needed community service [21]. Equal access to mental health care and psychosocial and community-based supports for Aboriginal people who are experiencing a suicide crisis or poor mental health is important because if community members are not accessing mental health services when they need to it is likely to result in poorer health and mental health outcomes in the short and longer terms [21]. The frequency with which participants described these poor experiences should be further explored and addressed by the local health services, with consideration for models that provide capacity for Aboriginal communities and mental health services to work together to identify and address systemic racism and to develop culturally competent models of mental health care.

Fourth, the impact of silence around suicide was a key theme. Participants described a lack of discussion about suicide generally, feeling ‘silenced’ and unable to reach out for help when in a suicide crisis or when grieving over the loss of a loved one to suicide and not knowing what to say to others who were struggling. Participants especially felt unable to discuss suicide because they recognised that their family and community had already experienced enough trauma and feared causing them further harm. However, participants also recognised the significant personal cost of staying ‘silent’, leaving them unable to share their grief with their support system, to make sense of their loss, to process their grief or to keep the memory of the deceased alive. Given the outcomes of this study, we suggest that ‘silence’ is a maintaining factor of grief and consequently may in itself be a risk factor contributing to higher rates of suicide in Aboriginal communities. For this reason, future research that looks at effective strategies to break the ‘silence’ and promote safe suicide discussions in families bereaved by suicide and the broader community may go some way to establishing better support for the bereaved and, importantly, could help prevent future suicides in Aboriginal communities.

Fifth, participants in this study spoke frequently about the long-lasting impact of the ‘ripple effect’ of suicide, including the ongoing cascade of negative events experienced by the family unit and the impact this had on their capacity to participate in vocational, educational and community activities. The ‘ripple effect’ following a death by suicide has received some attention in the published literature with respect to the broad and long-lasting impact of suicide on families and communities [22, 23]. The current study adds to the existing literature by highlighting that ‘ripple effects’ are apparent in Aboriginal communities and may have even more impact in these communities because of a combination of strong connections and interrelated relationships between and within Aboriginal communities. This means that the ‘ripples’ may be greater (because of the strong within- and cross-community connections), more intense (because of closer connections) and longer-lasting given the ongoing impact of inter-generational trauma, exposure to racism, discrimination and social disadvantage experienced by Aboriginal Australians.

Finally, the results of this study also suggest that the ‘ripple effect’ may play a role in both promoting and preventing suicide discussions in Aboriginal communities. Aboriginal participants who had been impacted by suicide reported strong attitudes about the importance of preventing future deaths and a strong belief that every Aboriginal community member has a responsibility to discuss suicide and to reach out. However, they simultaneously described a low level of confidence in their ability to discuss suicide and a sense that they are powerless to act to improve experiences of support when accessing mental health services, resulting in ‘silence’ described as both contributing to and perpetuating the cycle between these factors. It is therefore reasonable to conclude that future studies that aim to increase suicide discussions in Aboriginal communities must recognise the interrelatedness between the drivers of suicide discussions and focus on working with Aboriginal communities to build peoples’ confidence and capacity to discuss suicide and simultaneously improve access to culturally appropriate mental health services for Aboriginal communities. Furthermore, the inter-relationships between the perceptions and the experiences suggest that addressing either of these drivers in isolation may have the unintended effect of increasing rather than decreasing suicide risk in Aboriginal communities. Using a sociological lens [24] may be particularly useful in understanding how silence plays a role in the ripple effect and in what it means for Aboriginal communities; and it would also be valuable to explore the role that connection to culture may play in being protective against suicide at a community level [24–26].

On a local level, the findings from this study have helped to inform the work being implemented as part of the NSW Towards Zero Suicides initiatives (27) and that led by the Mental Health Service Closing the Gap committee. These groups are working on improving the cultural inclusiveness and safety of the Mental Health Service within these regions, including developing more accessible and community-focused points of contact for suicidal and mental health related crises. It will be important to investigate whether people’s experiences of mental health service contact changes over time and the degree to which it meets community needs more broadly.

### Limitations

While this study captured the views of Aboriginal participants across a large area in NSW, the findings must be interpreted within the context of its limitations. First, the study utilised a qualitative group methodology that relied on self-selection for participation and active group participation within the focus group. Therefore, it is possible that the views presented only reflect the perceptions of those who have been most affected by suicide or those who held the strongest views about the topic. Furthermore, due to the sensitivity of the topic and the potential community connections between participants, it is possible that some participants may have felt unable to participate openly in the focus group discussion, for example, because they did not see themselves as an expert or someone who could talk with authority about mental health or suicide, or they may have had concerns about confidentiality. Finally, whilst three focus groups were held across a diverse geographical region, this study was exploratory in nature and is unlikely to have captured the different perspectives of all members of the different communities involved in the study.

## Conclusion

The current study found that ‘silence’ around suicide, the ‘ripple’ effect of trauma and feeling ‘powerless’ to prevent suicide were important drivers of barriers to discussing suicide and seeking assistance for Aboriginal people when experiencing a suicide crisis. The findings highlight the interrelatedness between individual and community-level barriers of fear and shame and system-level considerations that result in Aboriginal people not receiving appropriate mental health care. For this reason, we believe that to be effective future suicide prevention initiatives must address both the ‘silence’ around suicide and the ‘ripple effect’ of trauma that already exists within Aboriginal communities and seek to improve access to culturally competent mental health care for Aboriginal communities. We also suggest that caution must be exercised in attempts to address these factors independently, as this may unintentionally result in an increased risk of suicide. Instead, a holistic and community-led approach is required.

## Data Availability

The data generated and analysed for this has been stored in a restricted file share held by the Hunter New England Local Health District. The data is not available for public use and will not be shared due to privacy, confidentiality and cultural reasons.

## Appendix

See Table

**Table 1:** Final qualitative code book

1. Attitudes	Description
Importance of SD_negative*	Discussions not in support of SD in community. ‘How important is it to talk about suicide in the community?’
Importance of SD_positive*	Indicating support for the need for SD in community. ‘How important is it to talk about suicide in the community?’
IND_fear of someone dying by suicide	Positive statement towards discussing suicide driven by fear of losing someone
IND_lived experience of losing someone to suicide_death	Positive towards discussing suicide based on the background of losing someone and wanting to prevent this grief for other individuals, families and communities
IND_suicide is an increasing issue	Positive statement towards suicide discussions given it is a growing issue
2. Barriers-Suicide Discussions	‘Potential barriers to discussing suicide in Aboriginal communities’ Categories consistent with the published literature
Accessing mental health services*	Statement suggesting previous contact with mental health services
ACCESS_negative	Statement suggesting contact with mental health service was negative
Culturally relevant mental health treatment*	‘Lack of the awareness of effective mental health treatments for Aboriginal people’
B_IND_negative general	Statement suggesting a general negative experience with accessing a mental health service
B_IND_perceived racism	Statement suggesting a negative experience due to perceived racism by health staff
B_Lack of awareness of cultural protocols*	Lack of awareness of the role of cultural processes and protocols when delivering mental health services for Aboriginal people
B_Lacked an awareness of Aboriginal mental health*	Barrier consistent with ‘lacked an awareness of Aboriginal conceptualisations of mental health’
B_No Aboriginal staff*	‘Lack of integration of Aboriginal clinicians and other Aboriginal colleagues into the mental health care of Aboriginal people’
ACCESS_positive experience*	Statement providing positive feedback about access to mental health
F_culturally relevant mental health treatment*	Mental health staff demonstrated an ‘awareness of effective mental health treatments for Aboriginal people’
F_Lack of awareness of cultural protocols*	Mental health staff demonstrated an awareness of the role of cultural processes and protocols when delivering mental health services for Aboriginal people
F_Lacked an awareness of Aboriginal mental health*	Mental Health staff demonstrated an ‘awareness of Aboriginal conceptualisations of mental health’
F_No Aboriginal staff*	‘Integration of Aboriginal clinicians and other Aboriginal colleagues into the mental health care of Aboriginal people was evident’
B- IND_Workforce issue	Indicating that aspects of the workforce present a barrier to discussing suicide
B_IND_workforce_need for self-care	Indicating need for self-care in the workforce
B_IND_workforce_training	Indicating workforce skill as a barrier to discussing suicide
B-IND_workforce_funding	Funding as a contributor to workforce issues
B-IND_workforce_time	Indicating time as a barrier
B-IND_workforce_training and development of Aboriginal community	Barrier recognising limited training to grow Aboriginal workforce
B-IND-workforce_cultural considerations	
B_Absence of Aboriginal-specific suicide prevention initiatives*	Statement suggesting there is a lack of Aboriginal-specific suicide prevention initiatives and/or over-reliance on adapting non-Aboriginal programs for implementation in Aboriginal communities
B_Other*	Other barriers identified by community
B-Fear of discussing suicide*	
B-Cause harm*	Statement that fear is driven by perception that SD may cause harm
B-IND_Fear _general	Statement describing a general fear of discussing suicide
B-IND_Fear of being removed from community	Statement supporting the belief that if unwell will be removed from community
B-IND_Feeling unable to provide appropriate information	Statement suggesting that feeling unable to support person if needed
B-Government policy*	Statement suggesting that government policies represent a barrier to discussing suicide
B-Powerlessness*	Statement supporting a perception of being powerless to provide support due to reason outside of their control
B-Racism experiences*	
B-Shame*	Barrier to discussing suicide is shame brought to individual or family
IND_Shame_ being labelled as womba	Statement suggesting that individuals with a mental health issue are not well supported by community members
IND_Shame_isolation of people with mental health issues in community	Statement suggesting that individuals with mental health concerns are isolated and/or distanced from community
IND_Shame_not feeling supported by community re SD	Statement suggesting that the individual does not feel supported by others in the community to have SD
IND_Shame_on family	
IND_Shame-experiences in education	
B-Suicide viewed as an individual issue*	Statement supporting that suicide is an individual choice and it is not the business of others to intervene

Deductive codes sourced from the literature are identified in the code book with the use of an Asterix (*) and inductive codes based on additional themes emerging from the consultations are identified by the letters IND

1.

## Abbreviations

Aboriginal: A collective term to refer to Aboriginal and Torres Strait Islander people
ASEW: Aboriginal social and emotional wellbeing
